# Insect Infestation Increases Viscosity of Biogenic Secondary Organic Aerosol

**DOI:** 10.1021/acsearthspacechem.3c00007

**Published:** 2023-04-25

**Authors:** Natalie R. Smith, Giuseppe V. Crescenzo, Allan K. Bertram, Sergey A. Nizkorodov, Celia L. Faiola

**Affiliations:** † Department of Chemistry, University of California, Irvine, Irvine, California 92697, United States; § Department of Chemistry, University of British Columbia, Vancouver, BC V6T 1Z1, Canada; ‡ Department of Ecology and Evolutionary Biology, University of California, Irvine, Irvine, California 92697, United States

**Keywords:** Monoterpene, sesquiterpene, herbivory-induced stress, plant stress volatiles, biogenic volatile organic compound emissions, aerosol particle mixing time

## Abstract

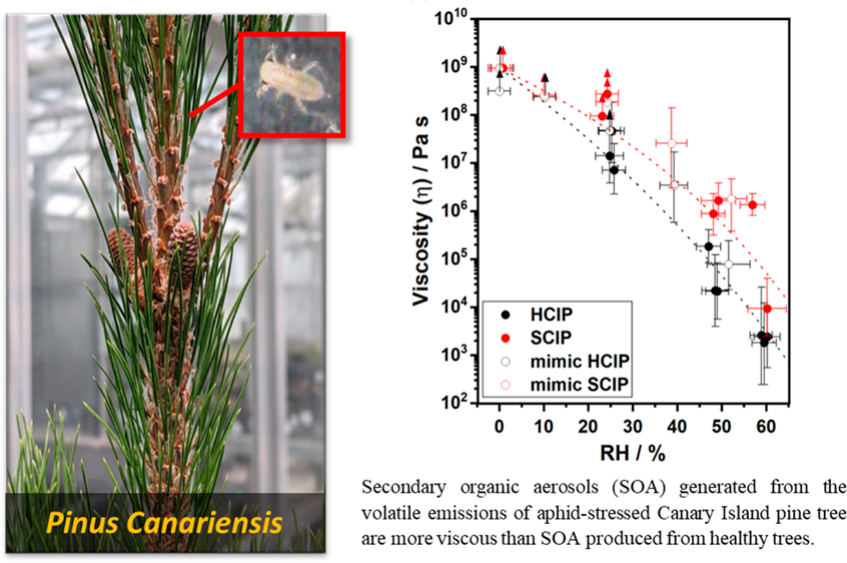

Plant stress alters emissions of volatile organic compounds. However, little is known about how this could influence climate-relevant properties of secondary organic aerosol (SOA), particularly from complex mixtures such as real plant emissions. In this study, the chemical composition and viscosity were examined for SOA generated from real healthy and aphid-stressed Canary Island pine (*Pinus canariensis*) trees, which are commonly used for landscaping in Southern California. Healthy Canary Island pine (HCIP) and stressed Canary Island pine (SCIP) aerosols were generated in a 5 m^3^ environmental chamber at 35–84% relative humidity and room temperature via OH-initiated oxidation. Viscosities of the collected particles were measured using an offline poke-flow method, after conditioning the particles in a humidified air flow. SCIP particles were consistently more viscous than HCIP particles. The largest differences in particle viscosity were observed in particles conditioned at 50% relative humidity where the viscosity of SCIP particles was an order of magnitude larger than that of HCIP particles. The increased viscosity for the aphid-stressed pine tree SOA was attributed to the increased fraction of sesquiterpenes in the emission profile. The real pine SOA particles, both healthy and aphid-stressed, were more viscous than α-pinene SOA particles, demonstrating the limitation of using a single monoterpene as a model compound to predict the physicochemical properties of real biogenic SOA. However, synthetic mixtures composed of only a few major compounds present in emissions (<10 compounds) can reproduce the viscosities of SOA observed from the more complex real plant emissions.

## 1 Introduction

Plants emit most of the total volatile organic compounds (VOCs) found in the atmosphere.^1^ Pine trees, in particular, have high emission rates of terpenes including 2-methyl-3-buten-2-ol, numerous isomers of monoterpenes (C_10_H_16_), and sesquiterpenes (C_15_H_24_) of various reactivities and structures.^1,2^ Terpenes undergo atmospheric oxidation, which produces low volatility and semivolatile species that condense to form secondary organic aerosol (SOA) particles. These SOA particles play a role in influencing climate, health, and visibility.^3^

Plant VOC emission profiles are highly complex, with over 1,700 different compounds identified across 90 plant families.^4^ Even emissions from an individual plant can contain up to 20–30 different terpenoid compounds.^5,6^ To add further complexity, plant VOC emission profiles change seasonally and diurnally, corresponding to changes in phenological and metabolic processes over long and short time scales.^7^ Additionally, plant VOC emission profiles shift in response to environmental and biotic stressors such as temperature extremes and insect infestation.^8−11^ For example, plant stress induced by insect herbivory increases emission rates of sesquiterpenes from pine trees which are produced through a biochemical defense pathway that functions in plant defense processes.^8,12^ Plants are exposed to longer and more severe periods of stress due to climate change.^13^ Specifically, insect infestations are increasing in frequency and duration as a result of increasing wintertime temperatures that reduce insect mortality.^14^ In a study by Bergström et al. (2014), it was estimated that plant VOC emissions induced by insect herbivory could account for 50% of all organic aerosol mass in Europe.^15^ In addition to altering the total amount of SOA produced, slight modifications to the VOC profile can result in significant changes to SOA chemical composition, mass yield, and volatility due to differences in reactivity and oxidation products between terpenoid compounds.^8,16^ Previous studies have demonstrated that chemical changes in the VOC profile that are induced by plant stress can alter SOA mass yields,^16^ chemical composition,^6,8^ and hygroscopicity.^17^ All of this suggests that plant stress will change other climate-relevant SOA properties, such as viscosity, but no study has directly measured changes in viscosity between SOA generated from real healthy and stressed plant emissions.

Viscosity is an important physical property of SOA and is highly influenced by particle chemical composition.^18−21^ An increase in viscosity can lead to much slower diffusion rates of compounds within the particles, impacting particle growth^22,23^ and evaporation,^24^ gas-particle partitioning,^25,26^ heterogeneous chemistry,^27−31^ particle-phase photochemistry,^32−34^ and the ability of SOA particles to act as nuclei for ice particles.^35,36^ Smith et al. (2021) recently reported that synthetic mixtures of VOCs representing the volatile profile of healthy boreal forest (Scots pine) trees produce highly viscous photooxidation SOA (>10^7^ Pa s) at relative humidity <40% RH.^37^ Synthetic mixtures of VOCs representing the volatile profile of aphid-stressed trees produce SOA with an order of magnitude higher viscosity under the same RH conditions.^37^ This difference was attributed, at least in part, to the relative amount of sesquiterpenes used to generate the SOA, since sesquiterpenes produce SOA compounds with higher molecular weights and, therefore, higher glass transition temperatures and lower hygroscopicities.^37^ However, the viscosity of SOA produced from real pine tree emissions was not investigated, so it remains unclear if the increased viscosity observed from synthetic mixtures could be reproduced from a more environmentally relevant system, such as a real plant.

Sesquiterpenes likely play an under-appreciated role in SOA generation in regions with pine trees. Our previous work demonstrated that small increases in sesquiterpenes lead to measurable changes in particle viscosity in the laboratory, but there is also evidence of their importance from field observations.^37^ For example, Barreira et al. (2021) observed sesquiterpene oxidation products in a springtime hemiboreal forest with mass concentrations of 0.07 μg m^–3^ in gas phase and 1.6 μg m^–3^ in particles.^38^ They also reported lower volatilities of compounds in the particle phase during a sesquiterpene-dominated period compared to a monoterpene-dominated period.^38^ A substantial contribution of sesquiterpenes to hemiboreal SOA formation during spring suggests that both atmospheric measurements and models that focus on monoterpene oxidation may overlook a potentially large fraction of SOA particulate mass, missing important implications for climate-relevant SOA properties.

Previous studies investigating the properties of real plant SOA have focused on boreal forest trees such as Scots pine,^37,39^ due to their large geospatial abundance in the Northern Hemisphere.^8,40,41^ However, VOC profiles between pine species can be very different, so it is important to expand SOA studies to other types of prominent pine trees. There are few works investigating the chemical and physical properties of SOA generated from VOC emissions of plants commonly used in landscaping which are becoming increasingly prevalent with the expansion of urban greening programs. *Pinus canariensis* is a subtropical conifer species native to the western Canary Islands, off the coast of North Africa, leading to its common name Canary Island pine. This species grows well in Mediterranean climate and is frequently used in landscaping throughout California due to their drought and thermo-tolerant properties.^42,43^ The prevalent abundance of Canary Island Pines in Southern California makes it an important plant species to study for improved understanding of plant–atmosphere interactions in heavily populated areas such as the Greater Los Angeles region.

The goal of this work is to compare the viscosity of SOA particles generated from photooxidation of VOCs emitted by healthy and aphid-stressed Canary Island pine trees. This project builds off previous laboratory studies that reported higher SOA viscosity from stressed pine SOA compared to healthy pine SOA using synthetic VOC mixtures to represent healthy and stressed boreal pine emissions,^37^ but it takes it one step further using real plant emissions that are much more complex than synthetic mixtures and contain VOCs that cannot be purchased from commercial chemical suppliers. Stressed plant SOA is expected to have a greater viscosity than healthy plant SOA due to an increase in emissions of sesquiterpenes that may lead to high molecular weight and low volatility compounds. In this study, Canary Island pine trees (*Pinus canariensis*) were chosen as the VOC source to generate *real* healthy Canary Island pine (HCIP) and aphid-stressed Canary Island pine (SCIP) SOA. The SOA was generated by photooxidation of the VOCs in an environmental chamber. To our knowledge, this study is the first of its kind investigating the viscosity of Canary Island pine tree SOA and will be key in understanding the influence of climate change on the complex relationship between plant emissions, aerosols, and climate.

## 2 Experimental Methods

### 2.1 Tree Enclosure

Canary Island pine saplings (*Pinus canerienesis*) were used as the sole source of VOCs for the experiments outlined below. *Pinus canariensis* trees were obtained from Shadetree Nursery (Irvine, CA) and transported to the University of California, Irvine (UCI) greenhouse, where they were grown under ambient light and temperature conditions. The trees were donated to the nursery by a local developer in 15-gallon pots when they were approximately 5–6 feet in height and 3 years old. The Canary Island Pines serve as a reasonable model system for studying the aerosol chemistry of complex VOC mixtures that are dominated by α- and β-pinene, which is characteristic of many coniferous trees including most pines.^44^ Each pine tree was kept in a 15-gallon pot at the UCI greenhouse, watered at least weekly, and received no fertilizer supplements within the time frame of this study. The experiments discussed here were conducted when the trees were approximately 4 years old, still in 15-gallon pots.

Experiments were conducted using one plant at a time. One day before an experiment, a single plant was carefully transported from the greenhouse to the laboratory, where the environmental chamber was located. This provides adequate time for emission recovery after the transport process because jostling can result in temporary (up to 24 h) elevation in emission rates.^45^ An LED full spectrum grow lamp (Spider Farmer, SF1000, 1 m^2^ footprint) was used to provide light for the plant while it was in the laboratory. To capture VOCs emitted by the plant, a 2 m^3^ Teflon plant enclosure was used to contain it. The plant enclosure was hermetically sealed on all sides except the bottom, which was zip-tied at the base of the tree trunk, excluding the pot of soil. The pot of soil was not included in the plant enclosure to remove any contribution of VOCs coming from air–soil exchange, which is not the focus of this study. Clean humidified air (scrubbed of VOCs and particulate matter, but not scrubbed of CO_2_) flowed into the plant enclosure at a rate of 4.5 L min^–1^. A short piece of PTFE tubing was used at an outlet port of the plant enclosure followed by a heated stainless-steel tube (50 °C) attached to a Teflon diaphragm pump (N9 KP18, M&C), which actively pulled air (containing the emitted VOCs) from the plant enclosure. After the plant was conditioned to the air flow conditions for 1 h, the output of the pump was directed into the 5 m^3^ environmental chamber,^37,46^ with the lights off at a rate of 3.5 L min^–1^.^37,46^ The chamber loading continued for 10–24 h until it contained at least 30 ppb monoterpenes, monitored continuously by a proton-transfer-reaction time-of-flight mass spectrometer (discussed below). The UV-B lights inside the chamber were off during the chamber loading period and not turned on until SOA generation was initiated after injection of oxidant. While 30 ppb is higher than typical levels of terpenes observed in ambient air (normally <1 ppb), 30 ppb was the minimum mixing ratio required to produce enough aerosol mass for the viscosity experiments.^47−49^

### 2.2 PTR-ToF-MS

A proton-transfer-reaction time-of-flight mass spectrometer (PTR-ToF-MS; Ionicon model 8000) with H_3_O^+^ as the reagent ion was used to monitor the VOC mixing ratio inside the 5 m^3^ environmental chamber. Mass calibration for the PTR-ToF-MS was performed using *m*/*z* 21.0226, H_3_^18^O^+^; 33.9941, ^18^O^16^O^+^; and 39.0332, (H_2_^18^O)H_3_^16^O^+^. The gas-phase abundance of monoterpenes (*m*/*z* 137) and sesquiterpenes (*m*/*z* 205) were monitored over time. When the total monoterpene mixing ratio reached approximately 30–80 ppb (after 10–24 h of loading the chamber) the pine enclosure was disconnected from the environmental chamber and VOC injection was stopped.

### 2.3 TD-GC-MS

Prior to aerosol generation via photooxidation, cartridge samples (Markes, Tenax TA and Carbograph multibed stainless steel adsorbent cartridges) were collected from the aerosol chamber at a sampling flow rate of 450 cm^3^ min^–1^ for 5 min. Two separate cartridges were independently collected for each experiment to help reduce the measurement uncertainties. This sample collection was performed following the injection of VOCs into the chamber and prior to the addition of oxidant at the heated outlet (50 °C) of the chamber. A thermal-desorption (TD-100 XR: Markes International) gas chromatograph-mass spectrometer (Agilent 7890B Gas Chromatograph, Agilent 5975 Mass Spectrometer) equipped with an HP-5 (30 m × 320 μm × 0.25 μm, Agilent) column was utilized offline to measure the initial mixing ratios of individual VOC isomers (primarily monoterpenes, oxygenated monoterpenes, and sesquiterpenes) that were present in the chamber prior to SOA generation. The standards used to generate calibration curves are listed in the Supporting Information (Table S1). VOCs were identified by their mass spectral patterns, which were cross referenced with the NIST mass spectral database and actual mass spectra obtained from the standards. Only compounds that had a mass spectral match quality ≥80 compared to the reference spectra from the NIST database were included in our analysis. In cases where compounds had match qualities ≥80 compared to reference spectra but were identified as compounds other than those listed in Table S1, which we had calibration curves for, proxy compounds were used for quantitation instead. For example, α-pinene was used for quantitation of monoterpenoids and oxygenated monoterpenes that we did not have specified standards for. For sesquiterpenes other than those listed in Table S1, which had molecular weight of 204 and match qualities ≥80 compared to NIST reference mass spectra, the calibration curve obtained from β-caryophyllene was used as a proxy standard for quantitation.

### 2.4 ToF-AMS

The chemical composition of SOA particles was monitored using an online time-of-flight aerosol mass spectrometer (ToF-AMS, or AMS for short; Aerodyne, Billerica, MA, USA) operated in V-mode.^50^ Particles were vaporized at 600 °C and ionized using electron impact ionization at 70 eV. The AMS data were processed using Squirrel, version 1.62A for unit mass resolution (UMR) data and Pika, version 1.22A for high-resolution peak fitting. The improved-ambient method mentioned in Canagaratna et al. (2015) was used to generate elemental ratios for the experimental data, including O:C and H:C ratios.^51^

### 2.5 SOA Generation

A diagram of the SOA generation setup is shown in Figure S1, along with a photograph of a plant used to load the chamber with VOCs. The chamber was operated in a batch mode. After the TD-GC-MS cartridges were collected, 45 μL (2 ppm) of aqueous H_2_O_2_ (30 wt %, Fisher Scientific) was injected into the chamber through a separate heated inlet (50 °C). The chamber conditions ranged from 30 to 85% RH and 21–23 °C depending on the experiment. The bank of UV-B lights was turned on to initiate photooxidation of the VOCs, and they were allowed to react for 2 h. The OH steady-state concentration in the chamber was previously reported as 1.4 × 10^6^ cm^–3^ using the same approach (the exact value depends on the total VOC reactivity, but it is always of the order of 10^6^ cm^–3^).^37^ No seed particles were used in these experiments in order to avoid interference during the viscosity measurements, in which a common inorganic seed such as ammonium sulfate would result in a core–shell morphology that would be difficult to probe with the poke-flow technique. Particle mass concentration was measured by a scanning mobility particle sizer (SMPS; TSI 3080) equipped with a condensation particle counter CPC; TSI 3775). A wall loss correction with an effective overall mass concentration loss rate constant of *k*_w_ = 0.0028 s^–1^ was applied to the data set; an example of this correction is shown in Figure S2. (This wall-loss rate constant measured previously at 50% RH as part of routine chamber characterization tests; we have not corrected for the RH dependence of the rate constant when calculating SOA yields listed in Table 1.) A single nucleation event occurred for all SOA generation experiments, indicating particle formation only occurred at the onset of VOC addition from the Canary Island pine trees, a sample “banana plot” for HCIP1 is shown in Figure S3. SOA particles were then collected onto hydrophobically coated glass slides on a nonrotating micro-orifice uniform deposit impactor (MOUDI; MSP Corp. model 110-R). Only a single stage 8 was used, which is designed for 0.18–0.32 μm particles under normal MOUDI operation, but a wider range of particle sizes was likely collected because all the remaining MOUDI stages were removed. The flow rate during MOUDI operation was 30 SLM. The SOA samples were placed in a protective plastic enclosure, hermetically sealed in plastic bags with a vacuum food sealer, and stored in a −20 °C freezer until they were shipped on ice for viscosity analysis.

**Table 1:** Experimental Conditions for SOA Particles Generated in the Chamber from Different Canary Island Pine Trees (1–5)^a^

Pine tree ID	RH (%)	MT_i_^b^ (ppb)	OMT_i_^b^ (ppb)	SQT_i_^b^ (ppb)	ΔSOA^c^ (μg m^–3^)	ΔVOC_noOMT_^d^ (μg m^–3^)	ΔVOC_allOMT_^d^ (μg m^–3^)	SOA Yield^e^ (%)
HCIP1	55	29.64	0.18	0.02	56	150	161	35–38
HCIP2	54	31 ± 3	0.14 ± 0.02	0.06 ± 0.01	71	155	156	45–46
HCIP3	35	23.8 ± 0.1	0.19 ± 0.03	–	72	118	127	57–61
SCIP4	84	26 ± 1	0.66 ± 0.07	0.6 ± 0.2	112	132	138	81–85
SCIP5	60	88.87	0.27	1.89	93	274	276	34

a The VOC concentration used was the total VOC contribution from terpenes monoterpene (MT_i_), oxygenated monoterpene (OMT_i_), and sesquiterpenes (SQT_i_) identified using GC-MS. HCIP abbreviation designates healthy Canary Island pine trees, and SCIP designates stressed Canary Island pine trees.

b MT_i_, OMT_i_, and SQT_i_ refer to initial mixing ratio measured in the chamber before oxidation was initiated.

c SOA mass concentration was corrected for particle wall losses (an example is shown in Figure S2).

d ΔVOC upper and lower bounds were calculated assuming all or no OMT reacted, respectively. Detailed description is reported in the Supporting Information (S1).

e SOA mass yield calculation and description are reported in the Supporting Information (S1). The range corresponds to assumptions about reactivity of oxygenated monoterpenes (the lower limit assumes that all of them reacted, while the upper limit assumes that none of them did).

A summary of the experimental conditions for the final set of measurements is outlined in Table 1. Each experiment in the table refers to a single photooxidation chamber experiment using an individual pine tree as the SOA precursor (several preliminary experiments were initially performed to refine the experimental protocol; only the final experiments are included in Table 1). We note that it is much harder to achieve identical starting conditions for experiments with real plants compared to the experiments in which SOA is prepared from a single VOC or a synthetic mixture of VOCs. However, the experiments can still be classified as corresponding to either healthy or stressed Canary Island pine trees based on their VOC mixing ratio profile in combination with physical evidence of aphid-infestation on the trees. No physical evidence of aphid-herbivory was noted for the first three experiments, which are referred to hereafter as healthy Canary Island pines (HCIP1–3). For the last two experiments, aphid infestations were seen on the trees as shown in Figure S4. These aphid infested plants are referred to hereafter as aphid-stressed Canary Island pines (SCIP4 and SCIP5).

### 2.6 Viscosity Measurements

The poke-flow method was used to determine the SOA viscosity as previously described.^37,52,53^ Briefly, the poke-flow method relies on observing the flow of material under an optical microscope after deformation with a blunt object.^52,54^ In our study, a needle was used to poke a supermicron single particle sitting on the glass slide substrate (supermicron particles were naturally generated during SOA collection by impacting many submicron particles on the same spot on the glass slide followed by coagulation of the submicron particles). Removing the needle resulted in a visible hole and left behind a half-torus shaped deformation on the spherical cap supermicron particle (Figure S5). The poked particle was allowed to flow until the area of the hole (*A*) had recovered to one-quarter of the original area of the poke hole (1/4*A*). Given enough time, particles would recover to their original spherically capped geometry which is energetically favorable. The time of the 1/4*A* recovery is referred to as the experimental flow time (τ_exp,flow_). The viscosity of the SOA was determined from τ_exp,flow_ and fluid dynamics simulations, performed using the Microfluidics module within COMSOL Multiphysics.^52,53^ The simulations were similar to those previously reported in Smith et al. (2021).^37^ Under low relative humidity conditions, such as 0% RH, the HCIP and SCIP SOA did not visibly flow over the duration of the experiment. In this case, adjustments to the COMSOL Multiphysics model were made similar to those reported in Smith et al. (2021) and a lower limit to viscosity was obtained by assuming the SOA material flowed by ≤0.5 μm (the spatial resolution of the microscope) within the observation time in the experiments.^37^ The simulations required inputs of surface tension, slip length, density, and contact angle. The experimental flow times (τ_exp,flow_) were then used in the COMSOL Multiphysics fluid dynamic model with conservative upper and lower limits for the parameters outlined (Table S2). Conservative upper and lower limits for these parameters resulted in conservative upper and lower limits for the simulated SOA viscosities. Prior to poking the particles, the particles were conditioned to the surrounding RH for times ranging from 1 to 27 h. Within the uncertainties of the measurements, the viscosities were independent of the conditioning times (Figure S6). Particle evaporation tests were also performed for both systems to verify that there was no significant change in the size of the particles in the poke-flow experiments due to evaporation. For times up to 27 h, which is the maximum length of time of the poke-flow experiments, the change in the size of the particles due to evaporation was less than the uncertainty of the measurements (Figure S7).

Diffusion coefficients (*D*) of organic molecules within the SOA were calculated from the experimental viscosities (η, in units of Pa s) using the Stokes–Einstein equation:
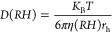
1where *K*_B_ refers to the Boltzmann constant, *T* represents the temperature in Kelvin, and *r*_h_ refers to the hydrodynamic radius which was assumed to be 0.38 nm for diffusing SOA molecules.^55^ The Stokes–Einstein equation provides reasonable estimates of diffusion coefficients of large organic molecules in SOA and proxies of SOA.^56−60^ The characteristic mixing time (*τ*_mixing_) within SOA particles in the atmosphere was then calculated from the diffusion coefficients using the following equation:
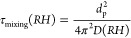
2where *d*_p_ represents the particle diameter. For this study, the particle diameter was assumed to be 200 nm, which corresponds to roughly the median diameter in the volume distribution of ambient SOA-containing particles and falls within the accumulation mode which can range from 100 to 1000 nm in diameter.^61−64^

## 3 Results and Discussion

### 3.1 Gas-Phase Composition

**Figure 1:**
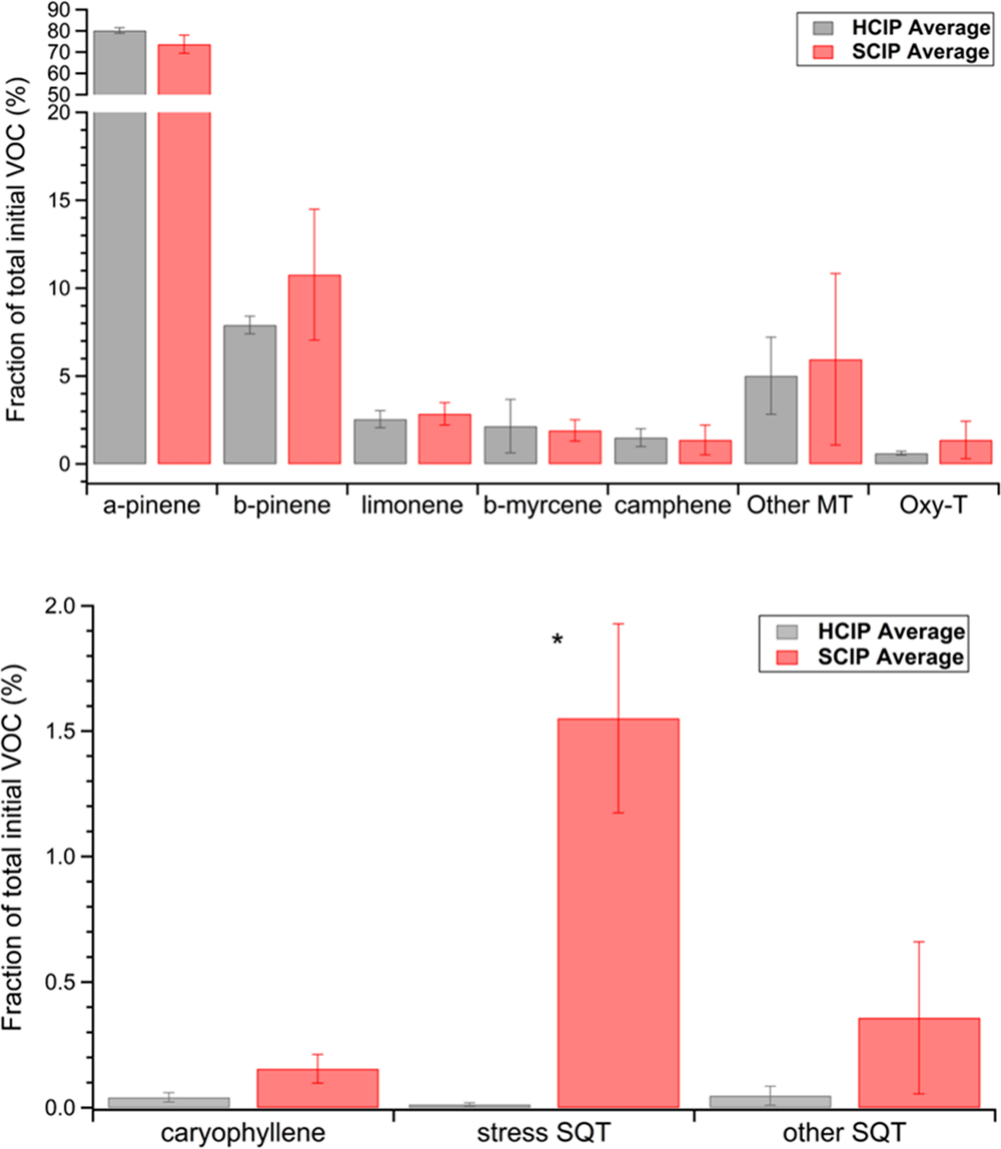
Average fraction of total initial VOC mixing ratio in the chamber at the start of the experiment for the healthy (HCIP1–3) and aphid-stressed (HCIP4–5) experiments. The Other MT category refers to other monoterpenoids. The Oxy-T category refers to oxygenated monoterpenes. The stress SQT category contains farnesene and germacrene. An asterisk (*) denotes statistical significance (*p* ≤ 0.01) with a Student’s *t* test (reported in Table S3). Error bars are standard error of the mean.

Average VOC composition profiles measured in the chamber for healthy tree experiments (HCIP1–3) and aphid-stressed experiments (SCIP4–5) are shown in Figure 1. In both healthy and stressed experiments, α-pinene contributed to approximately 70–80% of the total initial VOC mixing ratio in the chamber prior to photooxidation. This is expected as evergreen trees typically have high emission rates of α-pinene, as seen in studies investigating terpene concentrations in and above forested environments.^65,66^ The other monoterpenoid category (Other OMT) included monoterpenoids such as tricyclene (C_10_H_16_), Δ3-carene (C_10_H_16_), o- and p-cymene (C_10_H_14_), and isopropenyltoluene (C_10_H_12_). The oxygenated terpenes identified in the chamber are represented in the Oxy-T category and include the compounds bornyl acetate, verbenone, camphor, and borneol. Additionally, sesquiterpenes were identified, and the stress SQT category corresponds to the combined contribution of farnesene and germacrene D, which have previously been linked to volatiles induced by insect herbivory.^67^ The Other SQT category includes other cyclic sesquiterpenes with molecular weight 204, besides germacrene D or β-caryophyllene, such as copaene, cadinene, and muurolene. Importantly, monoterpene, OMT, and Oxy-T profiles were not significantly different between healthy and aphid-stress experiments. The only significant difference between the treatment groups was increased contribution of the stress SQTs (*p* < 0.01).

To aid in interpretation of the individual poke-flow experiments, it is helpful to discuss VOC profiles from the individual experiments (as opposed to averages of the different treatment groups). The VOC profiles for each individual experiments are listed in Table S3, and a brief discussion of the major differences is provided here. Emissions in all experiments contained a small amount of bornyl acetate, and all experiments except SCIP5 contained verbenone. In contrast, camphor and borneol were identified in only one experiment, SCIP4. The dominant stress SQT was germacrene D and α-farnesene for SCIP4 and SCIP5, respectively.

Evidence of the aphid infestation in SCIP experiments is provided in the Supporting Information. Figure S4a shows a close-up of the pine needles on SCIP4 that contain both live green aphids and aphid exuviae, which is the exoskeleton that an aphid sheds over the course of its life. Figure S4b shows a close up of a Petri dish containing the aphid exuviae and live aphids that had been shaken off from a group of pine needles on the tree. The live aphid (Figure S4c) was identified as a light green pine needle aphid (*Eulachnus brevipilosus*). Like all greenhouses, pest management is an ongoing struggle at the UCI greenhouse. To conduct the aphid exposure, we halted pesticide spraying in the room where the pines were stored and allowed the natural pests present in the greenhouse to colonize the plants. Plants were monitored regularly for signs of distress, but it is unclear exactly when the aphid infestation started. However, once the infestation was identified these plants were quarantined in a different room of the greenhouse to prevent induction of stress in the remaining trees through plant–plant communication through changes in volatile emissions. The minimum time between the first observation of aphids and the first stressed SOA experiment was roughly 1 week.

Our work is consistent with previous observations that insect infestations can alter the quantity and types of VOCs being emitted by the plants.^8,68^ Overall, the SCIP trees had a statistically significant increase in fractional contribution of stress SQT compared to the HCIP trees, as determined by using a Student’s *t* test with a significance threshold of *p* ≤ 0.01. Individual *p*-values from this test are reported in Table S3. The increase in stress SQT was attributed to the aphid infestation. The emission of farnesene and germacrene D, which comprise the stress SQT category, have been previously reported as indicators of stress-induced volatiles, specifically stress hormones produced by pine trees as a result of aphid herbivory.^67^ In a study by Helmig et al. (2007), β-farnesene and β-caryophyllene were the most abundant sesquiterpenes identified over a pine forest, which is consistent with the volatile profile for SCIP trees in this study, indicating that these stress sesquiterpenes are relevant for aerosol chemistry in the natural environment.^67^ Apart from the stress SQT category, all other terpene contributions were approximately similar between the HCIP and SCIP systems.

### 3.2 Measured Viscosity

**Figure 2:**
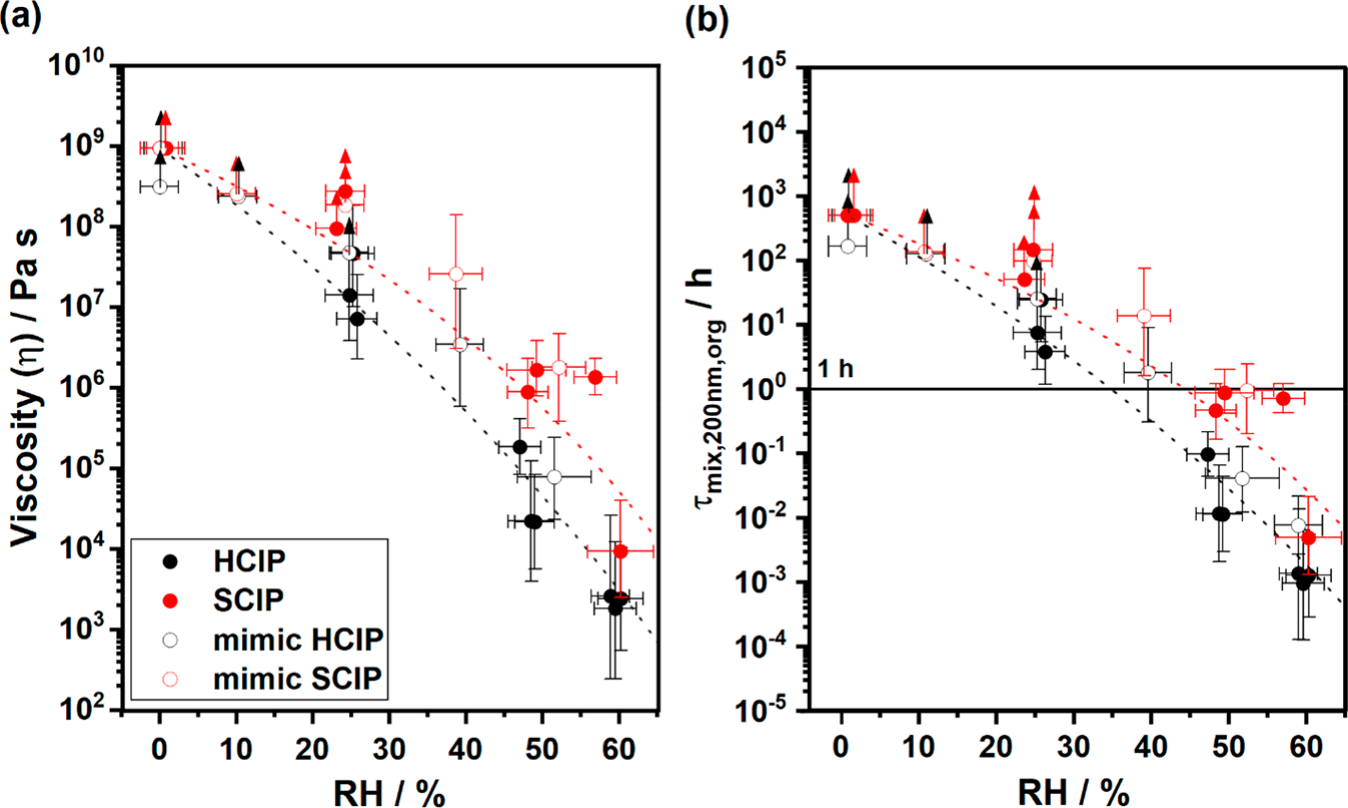
(a) Viscosity and (b) mixing times of organic molecules (τ_mix,200nm,org_) obtained from poke-flow measurements of healthy (*N* = 3) and stressed (*N* = 2) plant SOA as a function of relative humidity at room temperature (292 K). Error bars in the *y*-direction correspond to the upper and lower bounds of viscosity and mixing times determined from the range of input parameters used in the COMSOL simulations. The error bars in the *x*-direction correspond to the error in relative humidity from the measurement of dew points using a chilled mirror hygrometer. Upward arrows correspond to lower limits, and downward arrows correspond to upper limits. Open black circles (mimic HCIP SOA; *N* = 3) correspond to data from Smith et al. (2021) and Maclean et al. (2021);^37,39^ red open circles (mimic SCIP SOA; *N* = 3) correspond to data from Smith et al. (2021).^37^ The dashed lines intended to guide the eye of the reader are based on an Arrhenius mixing rule for the experimental data obtained in this study, and do not include the mimic HCIP and SCIP data.

The RH-dependent viscosities of the SOA particles generated from the healthy trees (HCIP1–3) are shown in red in Figure 2a and referred to as HCIP. Similarly, the two aphid-stressed tree SOA experiments (SCIP4 and SCIP5) are shown in black in Figure 2a and referred to as SCIP along with the healthy (mimic HCIP) and stressed plant mimic SOA (mimic SCIP) previously reported by Smith et al. (2021).^37^ As the relative humidity increases, the τ_exp,flow_ decreases corresponding to lowered viscosity due to the plasticizing effect water has on SOA.^18,69,70^ The experimental flow times (τ_exp,flow_) for the individual HCIP1–3 and SCIP4–5 SOA experiments are reported in Figure S8a,b, respectively. The relative humidity-dependent viscosity, derived from τ_exp,flow_ for the individual experiments (reported in Table 1), is shown in Figure S9.

HCIP and SCIP SOA viscosity values for the tree-emission SOA experiments are represented well by the respective mimic HCIP and SCIP viscosity values for the synthetic VOC mixture experiments (Figure 2a). The HCIP SOA viscosity was within experimental uncertainties of the mimic HCIP SOA viscosity reported by Smith et al. (2021)^37^ over the entire RH range investigated. The SCIP SOA closely followed the trend in viscosity as a function of RH for the mimic SCIP SOA system reported by Smith et al. (2021). The HCIP SOA particles (black) had lower viscosity than the SCIP SOA particles (red) between 0 and 60% RH, consistent with the trend reported by Smith et al. (2021). The difference in SOA particle viscosity between the two systems was largest at ∼50% RH where the SCIP SOA had an average viscosity between 10^5^ and 10^6^ Pa s compared to that of the HCIP SOA particles which had a viscosity between 10^4^ and 10^5^ Pa s and approximately an order of magnitude higher viscosity than for just α-pinene photooxidation SOA (10^3^–10^4^), as previously reported.^37^ At ≤10% RH, all systems had viscosities ≥10^8^ Pa s, which is greater than that of tar pitch which is highly viscous (10^8^ Pa s).

We note that SOA were prepared at different levels of RH in the chamber (Table 1), and the chamber RH could also affect the chemical composition and viscosity of the resulting SOA. However, we think the chamber RH effect is less important than the effect of the herbivory stress. Specifically, SCIP4 and SCIP5 SOA samples correspond to the highest (81–85%) and lowest (34%) RH in the chamber, respectively, but these two samples yield very similar viscosities (Figure S9).

It is remarkable that such large differences in viscosity (Figure 2a) are observed with such small changes in the VOC profile (Figure 1). Although the real and mimic SCIP SOA systems were dominated by monoterpenes (∼80%), they contained a slightly larger fraction of sesquiterpenes relative to their healthy counterpart systems. The mimic SCIP SOA had 20% more sesquiterpenes in the initial total VOC profile compared to the mimic HCIP SOA, and the real SCIP system exhibited a 3% increase in sesquiterpenes compared to that of the real HCIP system. The real SCIP SOA sesquiterpene profile was comprised of caryophyllene, germacrene D, α-farnesene, and a small fraction of “other” sesquiterpenes such as copaene, whereas the mimic SCIP SOA sesquiterpene profile consisted of only caryophyllene, isomers of farnesene, and valencene. This increased fraction of sesquiterpenes in the real stressed sample versus the real healthy sample could theoretically explain its higher SOA viscosity, due to generation of lower volatility species which would have higher glass transition temperatures, consistent with previous studies.^37,71^ However, the large changes in viscosity due to such small changes in relative sesquiterpene contributions could also suggest that the presence of other compounds in the mixture might be contributing to this effect. We cannot pinpoint the exact compounds producing this effect from the mixtures, but future studies can build off this work to systematically investigate the aerosol chemistry of the major monoterpenoids in the pine emissions reported here.

Chemical transport models often assume semivolatile compounds become well mixed within SOA particles on time scales less than 1 h.^3^Figure 2b shows mixing times of organics within a particle 200 nm in diameter as a function of RH for the four SOA systems. In all cases hereafter, references to mixing times refer to the mixing time within an SOA particle of size 200 nm. At ≤ 25% RH, all the SOA systems had mixing times greater than 1 h, contrary to assumptions in chemical transport models. For fast processes, equilibrium partitioning of SVOCs is the dominant growth pathway. However, if gas-particle equilibration time is slow, specifically for viscous particles, then the dominant SOA growth pathway will switch from equilibrium partitioning to kinetic uptake.^34^ In this case, models based on equilibrium partitioning will underpredict SOA mass concentration in the atmosphere.^72^ Shiraiwa and Seinfeld (2012) estimated that SOA mass concentrations could be incorrectly predicted by an order of magnitude using a kinetic flux model (KM-GAP) when organic aerosol is semisolid^73^ and would also result in incorrectly predicted SOA particle size.^74^ The mixing time of real biogenic SOA that get transported into the upper troposphere and lower stratosphere, which have very low humidity and temperature, could have mixing times ≥10 h. These longer mixing times should be considered when investigating long-range transport processes for these types of SOA because this would result in longer SOA lifetimes due to the reduced evaporation rates from particles as well as slower photodegradation rates observed within viscous SOA.^33,34^

### 3.3 Particle Phase Composition

The bulk aerosol mass spectra were dominated by organic compounds as expected since no seed particles were used in these experiments. The inorganic species such as nitrate, sulfate, and ammonium did not contribute to particle mass. The average elemental ratios were determined for experiments HCIP 2–3 and SCIP 4–5 (no AMS data were recorded for HCIP1.) The average O:C ratios (average ±1 standard deviation) over 30 min coinciding with peak SOA mass concentration in the chamber at the end of photooxidation were 0.55 ± 0.01 and 0.50 ± 0.01 for HCIP 2–3. The average O:C for the stressed plants SCIP4–5 were 0.50 ± 0.01 and 0.46 ± 0.01, respectively. Based on this result, the stressed plant SOA had slightly lower O:C than healthy plant SOA. The average H:C ratio was 1.63 ± 0.01 and 1.69 ± 0.01 for HCIP 2–3, respectively. The average H:C ratio was 1.46 ± 0.03 and 1.66 ± 0.01 for SCIP 4–5, respectively. Previous literature has reported a wide range of H:C and O:C ratios for SOA generated from different terpenes.^75^ The O:C values we report for Canary Island Pine SOA generated in a laboratory chamber are on the higher end of what has been reported previously for α-pinene (<0.4) and sesquiterpenes (<0.5).^51^ The SCIP SOA (which had slightly higher sesquiterpene contribution) had a lower O:C value. This could be related to differences associated with chemical properties of farnesene and germacrene D SOA, which have not been reported previously and comprised the stress sesquiterpenes in the SCIP experiments. It could also indicate slightly suppressed OH levels in the SCIP experiments due to farnesene scavenging of the OH radicals. This could occur because farnesene is an acyclic terpene with four double bonds, leading to a much higher OH reaction rate constant and increased likelihood to fragment upon oxidation compared to a cyclic sesquiterpene such as germacrene D.^8^ Furthermore, farnesene SOA yields are lower than other sesquiterpenes^6^ so more of the farnesene SCIP oxidation products would have remained in the gas-phase, effectively scavenging the OH out of the gas-phase and keeping it out of the condensed phase. Ylisirniö et al. showed that a sesquiterpene mixture containing farnesene isomers generated SOA at a yield ∼^1^/_3_ of that observed for α-pinene under the exact same conditions.^6^ This is in contrast to the many studies showing that cyclic sesquiterpenes, such as β-caryophyllene, have SOA yields approximately five times greater than that of α-pinene SOA.^16^ This is also consistent with results presented in Khalaj et al., who demonstrated a clear indirect relationship between increasing contribution of acyclic terpenes in a complex emission mixture and the resulting SOA yield.^76^

Interestingly, even the HCIP SOA had a higher O:C than typically reported from laboratory-generated monoterpene SOA. This is likely due to the contribution of other monoterpenoid compounds, including the oxygenated terpenoids, which produce more highly oxidized SOA than their nonoxygenated counterparts.^77^

**Figure 3:**
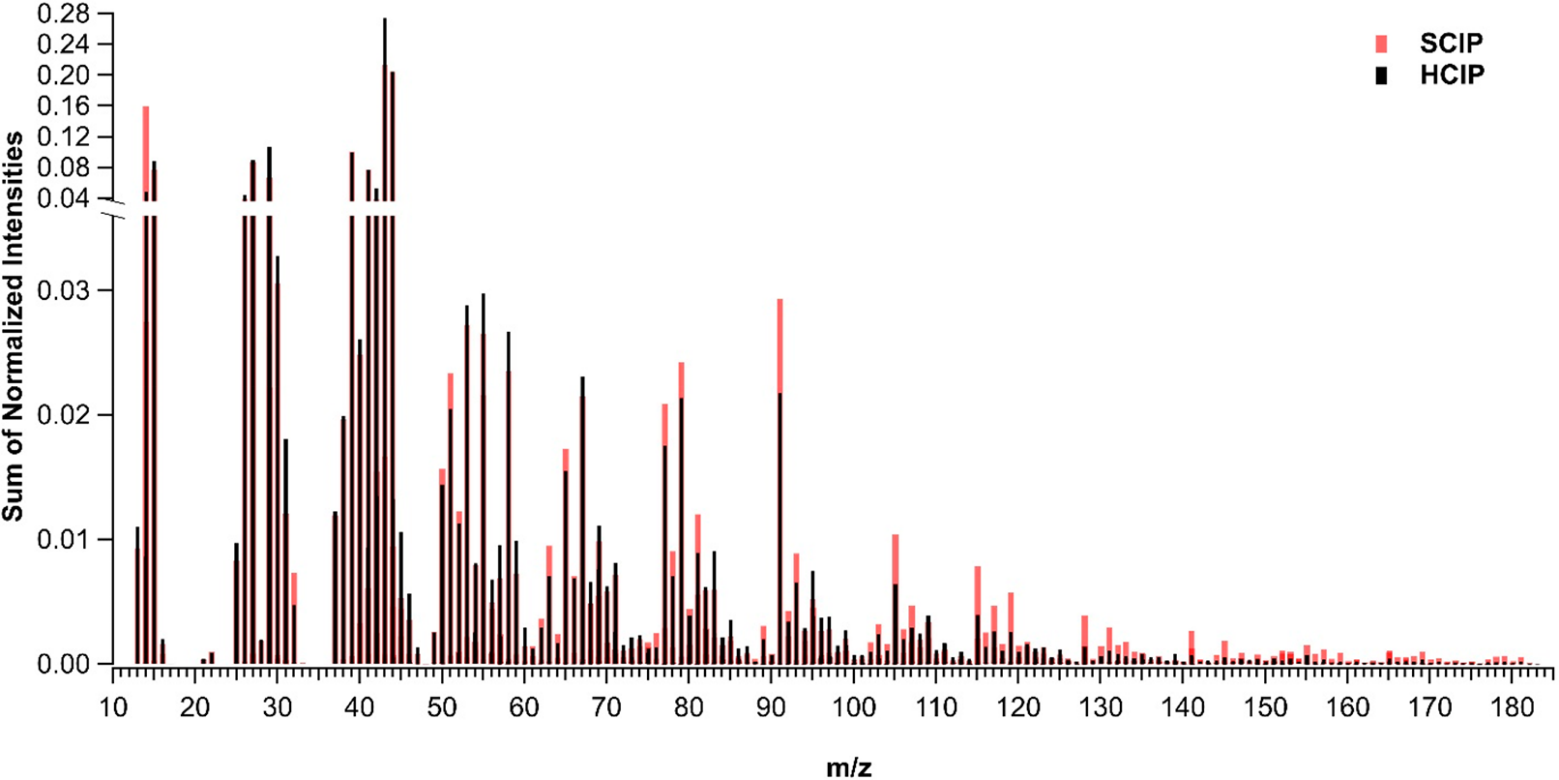
AMS mass spectra normalized to the sum of normalized intensities for HCIP2–3 (HCIP, black) and SCIP4–5 (SCIP, red).

**Figure 4:**
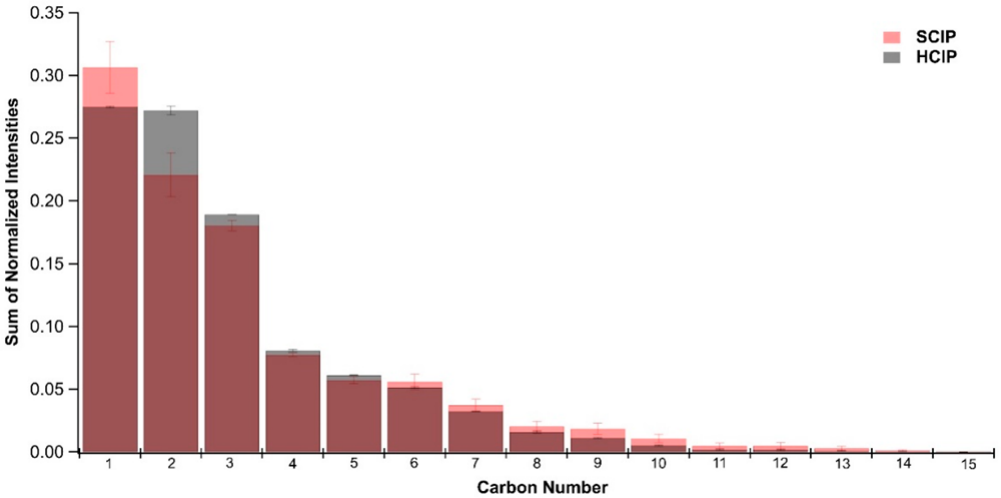
Sum of normalized intensities as a function of carbon number based on organic families for HCIP2–3 (HCIP, black) and SCIP 4–5 (SCIP, red) from AMS data.

The unit mass resolution of the average of healthy (HCIP 2–3, black) and stressed (SCIP 4–5, red) SOA determined from AMS data is reported in Figure 3 and was normalized to the sum of total intensities between the two systems for direct comparison. Figure 3 is only showing the unit mass resolution for the organic families consisting of CH (C_*x*_H_*y*_^+^), CHO (C_*x*_H_*y*_O^+^), and CHO_gt1_ (C_*x*_H_*y*_O_*z*>1_^+^) fragments. The relative contribution of CH, CHO, and CHO_gt1_ to HCIP was 48%, 38%, and 13%, whereas for SCIP CH, CHO, and CHO_gt1_ contributed to 51%, 36%, and 13%, respectively. The AMS data has low signal for ions with *m*/*z* above 180 because aliphatic compounds in SOA fragment extensively during vaporization and electronic impact ionization in AMS. The SCIP SOA had increased abundance of compounds with >10 carbon compared to the HCIP SOA (Figure 4). Our initial hypothesis was that the increased signal of compounds with >10 carbons for the stressed plant system is attributed to the increased abundance of sesquiterpenes identified in the initial VOC profile for the stressed SOA system, which would produce SOA products of higher molecular weight and therefore lead to overall higher particle viscosity compared to a healthy SOA system. However, a Student’s *t* test with a significance threshold of *p* ≤ 0.01 was used to determine statistical significance. The calculated *p*-values for the sum of normalized intensities between the HCIP and SCIP fragments with greater than 10 carbon atoms were not statistically significant (*p* = 0.4).

**Figure 5:**
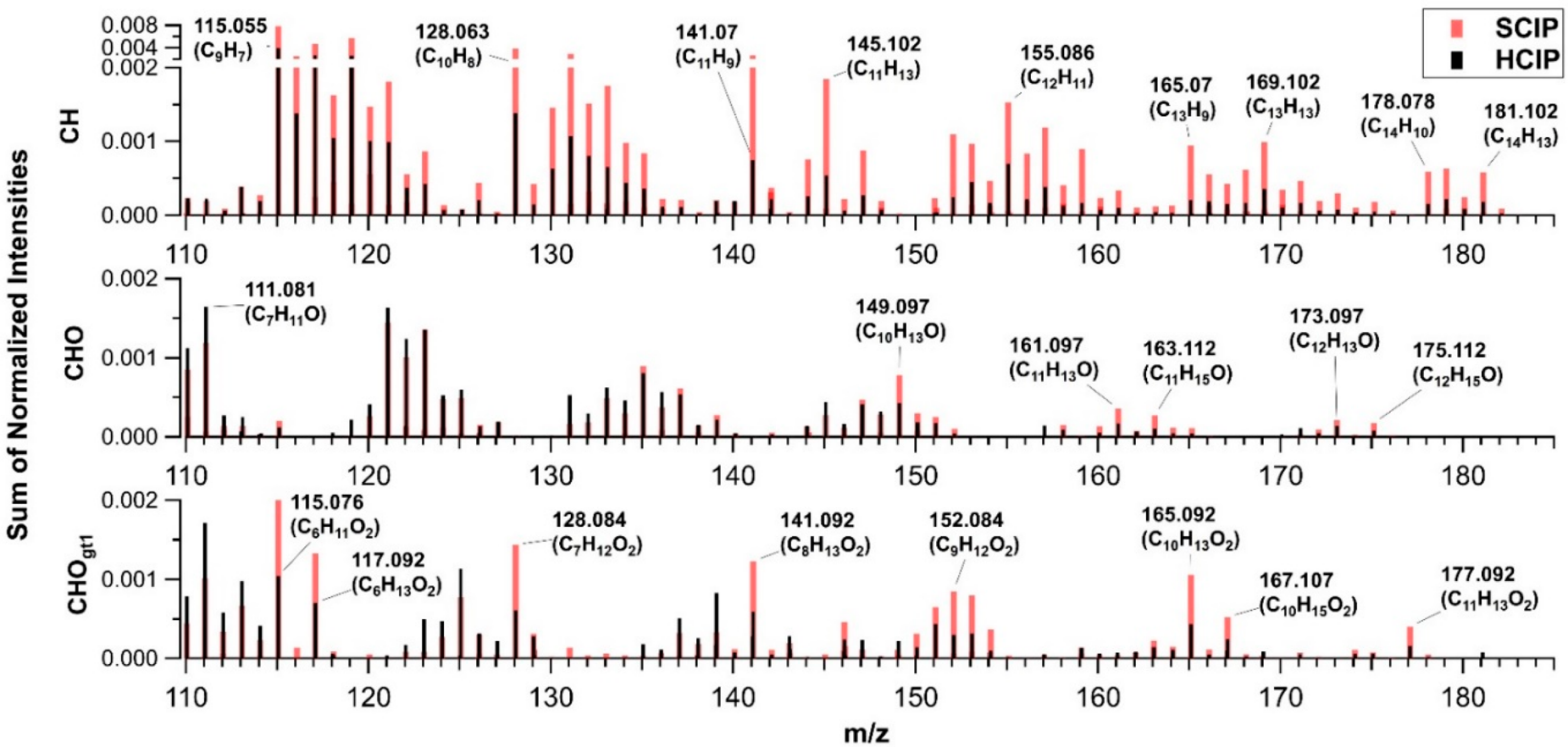
High-resolution mass spectra normalized to the sum of normalized intensities for HCIP2–3 (HCIP, black) and SCIP4–5 (SCIP, red) from AMS data, with respect to chemical family CH (C_*x*_H_*y*_^+^), CHO (C_*x*_H_*y*_O^+^), and CHO_gt1_ (C_*x*_H_*y*_O_*z*>1_^+^).

Figure 5 shows a breakdown of the organic ion fragments identified at *m*/*z* > 110 for the CH, CHO, and CHO_gt1_ families. Most of the ion intensity for organic fragments in both the HCIP and SCIP SOA was attributed to CH family fragments, as seen in Figure 5. Compared to the HCIP SOA, SCIP SOA had higher intensity in the CH and CHO_gt1_ families within this range compared to the HCIP and some minute differences within the CHO family.

Ions with >10 carbon atoms in larger quantities in the SCIP case are labeled in Figure 5. Ions such as C_14_H_13_^+^, C_13_H_13_^+^, and C_11_H_13_O_2_^+^ likely represent fragments of sesquiterpene oxidation products because they have >10 carbon atoms. If oligomerized monoterpene oxidation products were a major source of ions with >10 carbon atoms, they would have comparable peak abundances in both SCIP and HCIP mass spectra; however, they are clearly more abundant in the SCIP mass spectra. The SCIP spectra in Figure 3 also shows a large peak at *m*/*z* 91 (referred to as f91), which has been previously noted to be high for sesquiterpene SOA systems such as β-caryophyllene, a cyclic sesquiterpene with structure similar to germacrene D which dominated the SCIP4 SOA.^77^ In previous ambient and plant chamber studies, the *m*/*z* 91 peak in AMS data has been attributed to the tropylium ion (C_7_H_7_^+^).^77−79^ The increased abundance of *m*/*z* 91 for the SCIP compared to the HCIP can be explained by the higher fraction of sesquiterpenes in the initial VOC profile used to generate the SCIP SOA. The shift toward higher molecular weight fragments observed in the AMS data for SCIP is fully consistent with the higher viscosity for SCIP compared to HCIP SOA measured with the poke-flow method. While there is some variability in the emission profile within treatment groups, there is larger variation between the treatment groups as highlighted by the statistically significant increase in stress sesquiterpene emissions associated with the aphid-stressed plants. Therefore, the systematic difference in SOA viscosity between the treatment groups is attributed to the chemistry associated with the stress sesquiterpenes.

## 4 Atmospheric Implications

Plant stress can drastically alter the physical properties of SOA generated from VOCs emitted by plants. Even a relatively small increase in the fraction of sesquiterpenes emitted into the atmosphere leads to significant changes in the resulting SOA particle properties. This study reports novel humidity-dependent viscosity for healthy and aphid-stressed Canary Island pine tree SOA. We have demonstrated that real pine trees experiencing aphid herbivory generate SOA particles with higher viscosity compared to SOA generated from healthy trees of the same species. This confirms the results of our previous study in which SOA generated from proxy mixtures of VOCs representing the emission profile of real and aphid-stressed pine trees showed that including a small fraction of sesquiterpenes to a mixture of monoterpenes produced highly viscous particles at low relative humidity.

Our results have broad atmospheric implications because real SOA produced from trees have mixing times of organics >1 h under room temperature and <40% RH conditions, which has been previously suggested based on SOA generated from terpene mixtures but has not been verified with real tree SOA until now. Mixing times >1 h are significant since chemical transport models often assume mixing times shorter than 1 h when predicting SOA properties such as mass and size. In addition, this study verifies that a single monoterpene, such as α-pinene, cannot accurately represent the physicochemical properties of real biogenic SOA, and mixtures of terpenes containing a representative range of monoterpenes and sesquiterpenes should be used when investigating the fundamental properties of biogenic SOA systems in a laboratory setting. These findings are important as they suggest that in a changing environment where plant stress due to aphid herbivory is expected to increase, there will be higher emission rates of sesquiterpenes by plants which will lead to chemically and physically different SOA than is assumed for healthy or only monoterpene-containing SOA. More studies investigating the physical properties of stressed SOA are recommended to accurately assess their impact on climate and health. Since the results of this study closely follow those from Smith et al. (2021),^37^ it is also expected that the SCIP SOA in this study will exhibit liquid–liquid phase separation down to lower relative humidities compared the HCIP system which can impact processes such as cloud nucleation and long-range transport of the SOA, and this phenomenon will be investigated in future studies.

## Supporting Information Available

Description of sources of chemicals used (Table S1), a diagram of the experimental setup (Figure S1), an example of all-loss correction of SOA mass concentration in the chamber (Figure S2), particle size distribution as a function of photooxidation time (Figure S3), photographic evidence of aphid infestation of trees (Figure S4), description of SOA yield calculation (Section S1), optical images of particles taken during poke-flow experiments (Figure S5), list of COMSOL parameters used to calculate particle viscosity (Table S2), control measurements showing the lack of dependence of the measured viscosity on the exposure time (Figures S6 and S7), relative concentrations of VOCs emitted by trees (Table S3), experimental flow times from poke-flow experiments (Figure S8), and viscosity as a function of RH from poke-flow experiments (Figure S9) (PDF)
