# Supplementary material for: Insect Infestation Increases Viscosity of Biogenic Secondary Organic Aerosol
Source: ACS Earth Space Chem. 2023 Apr 25;7(5):1060–71. doi: 10.1021/acsearthspacechem.3c00007 (PMC10201571; doi:10.1021/acsearthspacechem.3c00007)
Supplement: Supplementary file 1 — sp3c00007_si_001.pdf [file sp3c00007_si_001.pdf]

Supplemental Information for:

Insect infestation increases viscosity of biogenic secondary organic aerosol

Natalie R. Smith[†], *Giuseppe V. Crescenzo*[§], *Allan K. Bertram*[§], *Sergey A. Nizkorodov*[†*], *Celia L. Faiola*[†, ‡, *]

† Department of Chemistry, University of California Irvine, Irvine, CA 92697, USA

§ Department of Chemistry, University of British Columbia, Vancouver, BC, V6T 1Z1, Canada

‡ Department of Ecology and Evolutionary Biology, University of California Irvine, Irvine, CA 92697, USA

VOC Standard	Chemical Structure	Purity	Source
α-pinene	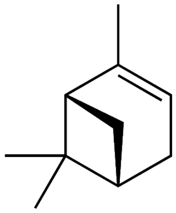	98 %	Acros Organics (CAS: 7785-26-4)
ß-pinene	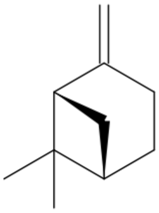	98 %	Acros Organics (CAS: 18172-67- 3)
ß-myrcene	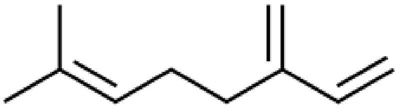	>75% (Contains 1000 ppm of BHT as inhibitor)	Aldrich (CAS: 123-35-3)
Δ^3-carene	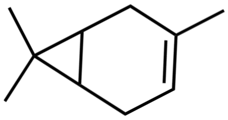	90 %	Aldrich (CAS: 13466-78- 9)
camphene	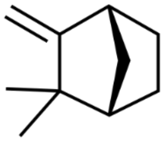	≥96%	Sigma Aldrich (CAS: 79-92-5)

limonene	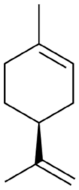	97% Stabilized	Alfa Aesar, (CAS: 5989-27-5)
ß-caryophyllene	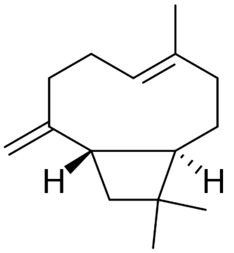	98.5%	Sigma (CAS: 87-44-5)
mix of farnesene isomers	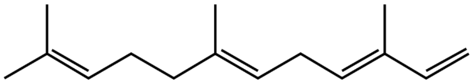 (α-farnesene) 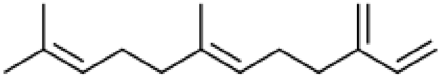 (β-farnesene)	stabilized (<0.10% α-tocopherol)	Sigma-Aldrich (Product#: W383902)

Table S1. Purity and sources of commercially available standards used to generate calibration curves for TD-GC-MS quantification.

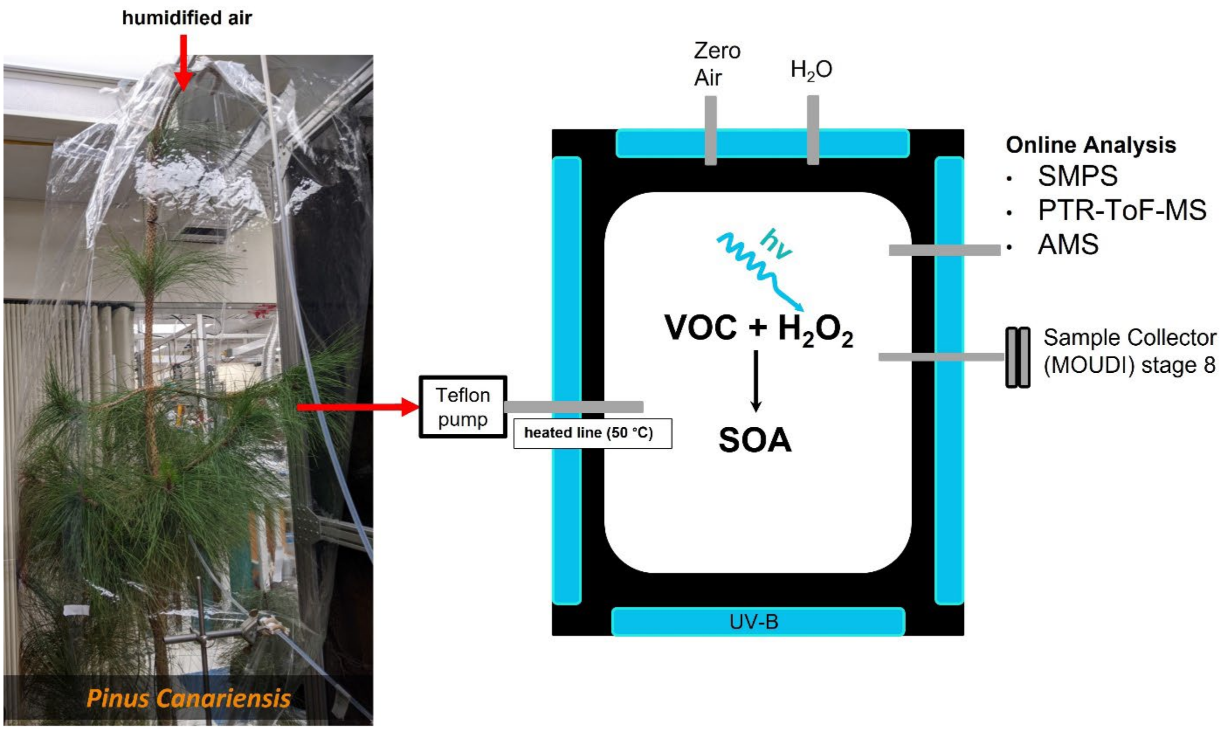

Figure S1. Experimental Setup for SOA generation in a 5 m^3 environmental chamber with 2 m^3 Teflon pine enclosure used a source of VOCs containing a Canary Island pine (*Pinus Canariensis*).

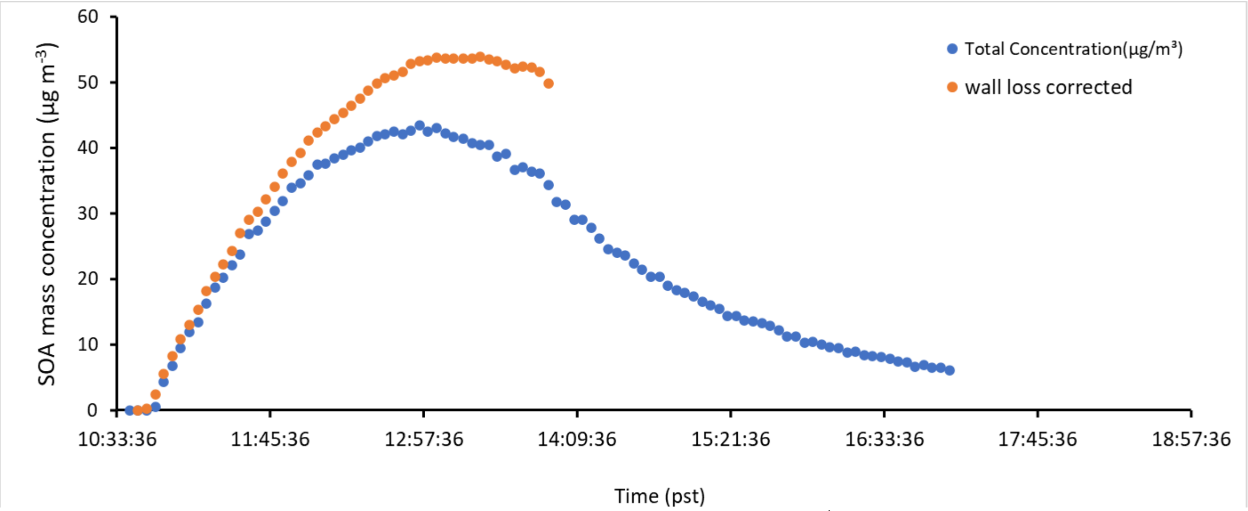

Figure S2. Example particle wall loss correction ($k_w = 0.0028\ s^{-1}$) applied to healthy plant SOA trial. Correction was only applied during the SOA formation phase, and not during the SOA collection phase when the particle loss became faster. Note that the wall-loss rate constant may depend on RH in the chamber, but the SOA yields reported in Table 1 do not take this dependence into account. Ideally, the wall loss rates should be measured for each experiment but doing this would mean losing a lot of SOA mass before it is collected. Therefore, we relied on previously measured wall loss rates for ammonium sulfate particles at 50% RH.

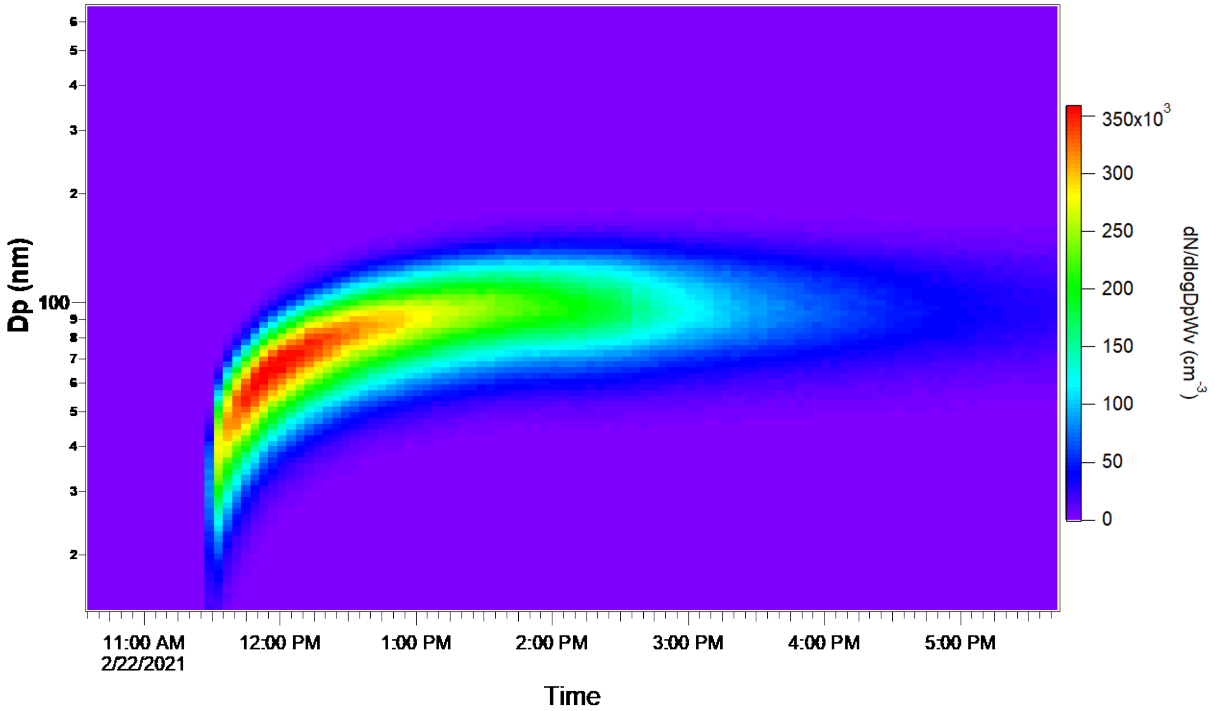

Figure S3. Particle formation as a function of time determined from SMPS data for SOA generated from HCIP1.

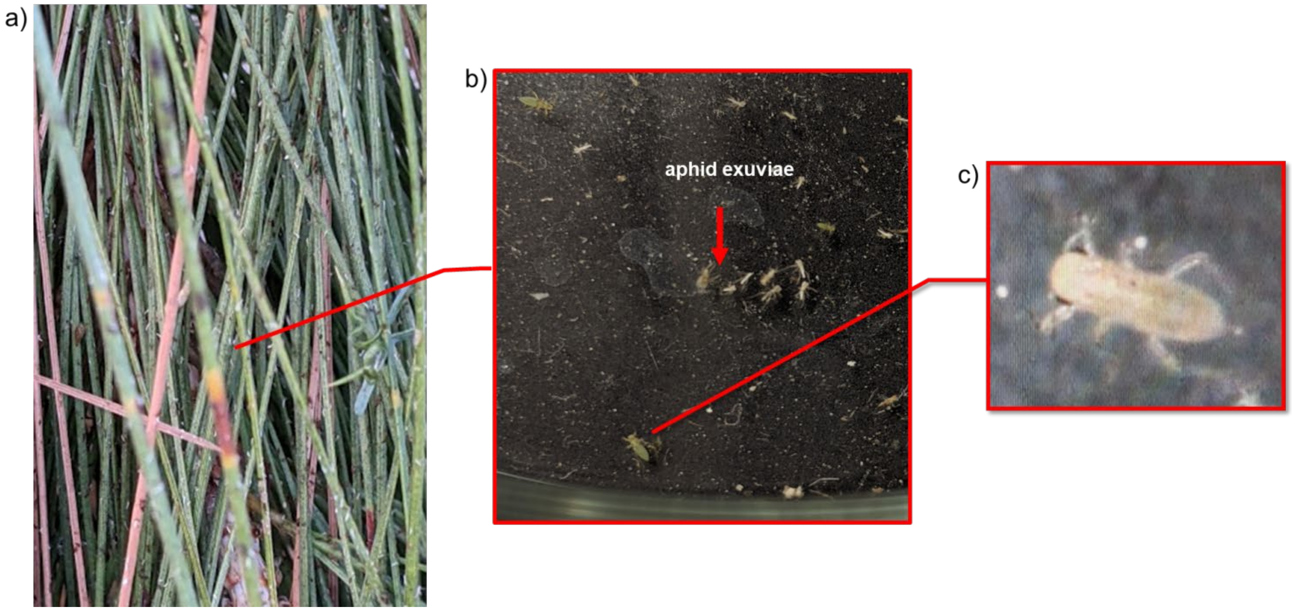

Figure S4. a) picture of aphid-infested tree (SCIP4). b) Sample of aphid and aphid exuviae (exoskeleton) collected from pine needles onto a petri dish. C) Close up of collected aphid, identified as a light green pine needle aphid (*Eulachnus brevipilosus).*

Section S1: SOA yield calculation

The SOA yield was calculated for each trial (CIP1-5) using the equation below.

$$SOA\ Yield\ (\%) = \frac{\Delta SOA}{\Delta VOC_{MT+OMT+SQT}} \text{ x } 100 \quad \text{(S.1)}$$

The change in SOA mass concentration (ΔSOA) was determined from the SMPS data and was corrected for particle wall losses (Figure S2). The SOA density in the SMPS was assumed to be 1.2 g cm^{-3} based on previous literature for biogenic SOA.[1–3] The change in VOC mass concentration (ΔVOC) was determined using a combination of PTR-ToF-MS data and TD-GC-MS data for the summed total mass concentration difference for monoterpene, oxygenated monoterpene, and sesquiterpene. The percent of reacted VOC was determined using PTR-ToF-MS data for total monoterpenes (*m/z* 137) and sesquiterpenes (*m/z* 205). As changes in oxygenated monoterpenes (OMT) could not be quantified, a range of reactivity was assumed for the OMT compounds. The low range for the SOA mass yield was determined assuming none of the OMT identified in the TD-GC-MS reacted away as a result of photooxidation whereas the high range for the SOA mass yield assumes that all (100%) of the OMT reacted away over the course of oxidation. To convert percent reacted monoterpene, oxygenated monoterpene and sesquiterpenes into mass concentration to be used in Equation S.1, the percent reacted monoterpene, oxygenated monoterpene, and sesquiterpene was multiplied by the total mass concentration of these individual terpene categories based on the TD-GC-MS data.

SOA yield results are reported in Table 1 in the main text. SICP4 had the highest yield followed by HCIP3, HCIP2, HCIP1, and SCIP5. The yield of α-pinene photooxidation was previously reported as 26.7 ± 2.5 % for SOA by generation through photooxidation with no seeds with a comparable mass concentration of SOA of 66.8 ± 6.0 μg m^{-3} at low RH.[4] Most of the trials in this study had SOA yields >30%, meaning that α-pinene alone cannot replicate the higher SOA

yield from real pine trees. Trials with high SOA yield correlated well with trees that had higher contribution of oxygenated monoterpene in the initial VOC profile in the chamber (Table S3), with SCIP4 having the largest followed by HCIP3, HCIP1, HCIP2, and HCIP5. However, it is worth noting that due to our assumptions with calculating the change in VOC mass concentration, it is possible there are some errors in our estimations of VOC yield and further investigation of how oxygenated monoterpene influences SOA yield from mixtures of biogenic volatiles is recommended. The high SOA yield for SCIP4 could also be attributed to increased fraction of germacrene D which is expected to have a higher yield due to more double bonds within the structure that are capable of oxidizing and forming lower volatility products. The yield for SCIP5 is not as high because the main stress SQT in this VOC system was farnesene, an acyclic sesquiterpene that could undergo fragmentation under photooxidation.[5] This is consistent with high-resolution mass spectra in the literature for farnesene photooxidation SOA made in an environmental chamber.[6] This suggestion is further supported by Faiola et al. (2019), who reported that the largest change in plant volatiles was farnesene in the stressed plant samples, however the plant volatile mixtures with more farnesene did not appreciably change SOA production from photooxidation (healthy 10.8-23.2% and stressed 17.8-26.8%).[7]

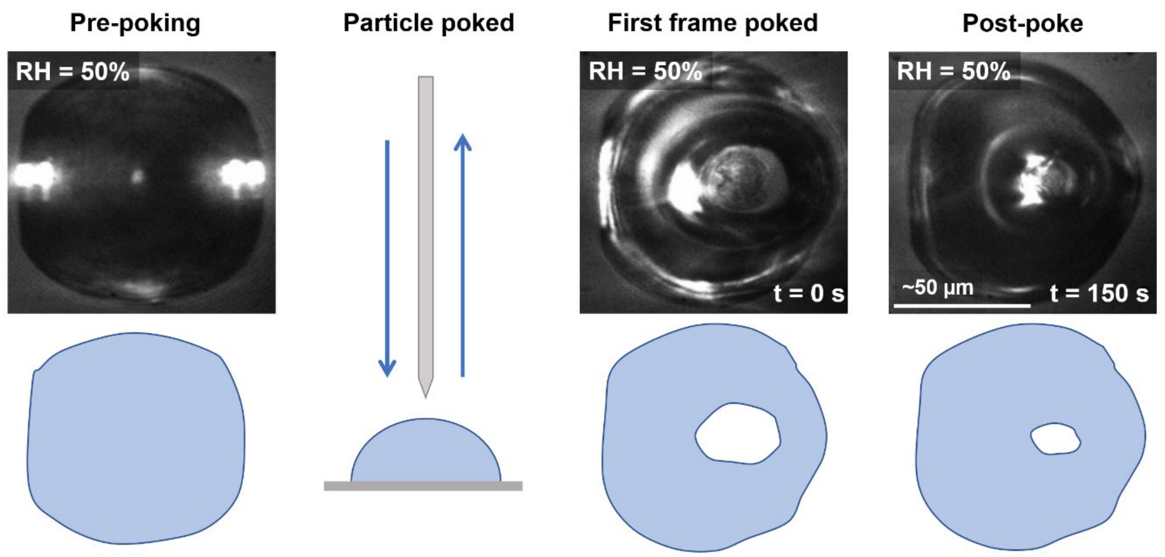

Figure S5: Optical images recorded throughout a poke-flow experiment. The images correspond to the poke-flow experiment of HCIP3 at 50% relative humidity. The scale bar shown corresponds to 50 µm.

Table S2: COMSOL parameters used for simulating the upper and lower limits of viscosity of the collected SOA by poke-flow at 292 K.

SOA type		Surface tension (mN m^{-1})	Slip length (m)	Contact angle (°)
HCIP1	Range of values	25.3^a-45^b	$5x10^{-9}$-$1x10^{-6\ c}$	45.7-60.7^d
HCIP2	Range of values	25.3^a-45^b	$5x10^{-9}$-$1x10^{-6\ c}$	56.2-61.7^d
HCIP3	Range of values	25.3^a-45^b	$5x10^{-9}$-$1x10^{-6\ c}$	53.4-58.8^d
SCIP4/SCIP5	Range of values	23.0^a-45^b	$5x10^{-9}$-$1x10^{-6\ c}$	22.5-35.1^d

[a] As a conservative lower limit to the surface tension of the SOA, the lowest surface tension of the pure liquid was used based on the distributions of molecules emitted by the saplings. Surface tensions were determined with the ACD/Labs Percepta Platform-PhysChem Module, retrieved from ChemSpider August 27, 2021.

[b] This upper limit is consistent with surface tension measurements of SOA at RH ≲65% RH and surface tensions reported for alcohols, organic acids, esters, and ketones, as well as surface tension measurements of water solutions containing SOA products.[8–11]

[c] Range based on measurements of the slip length of organic compounds and water on hydrophobic surfaces.[12–24]

[d] Contact angles determined by measuring the height and radii of individual droplets using a confocal microscope following the method of Ref. [25]. Note: the simulated viscosities depend only weakly on the contact angle.

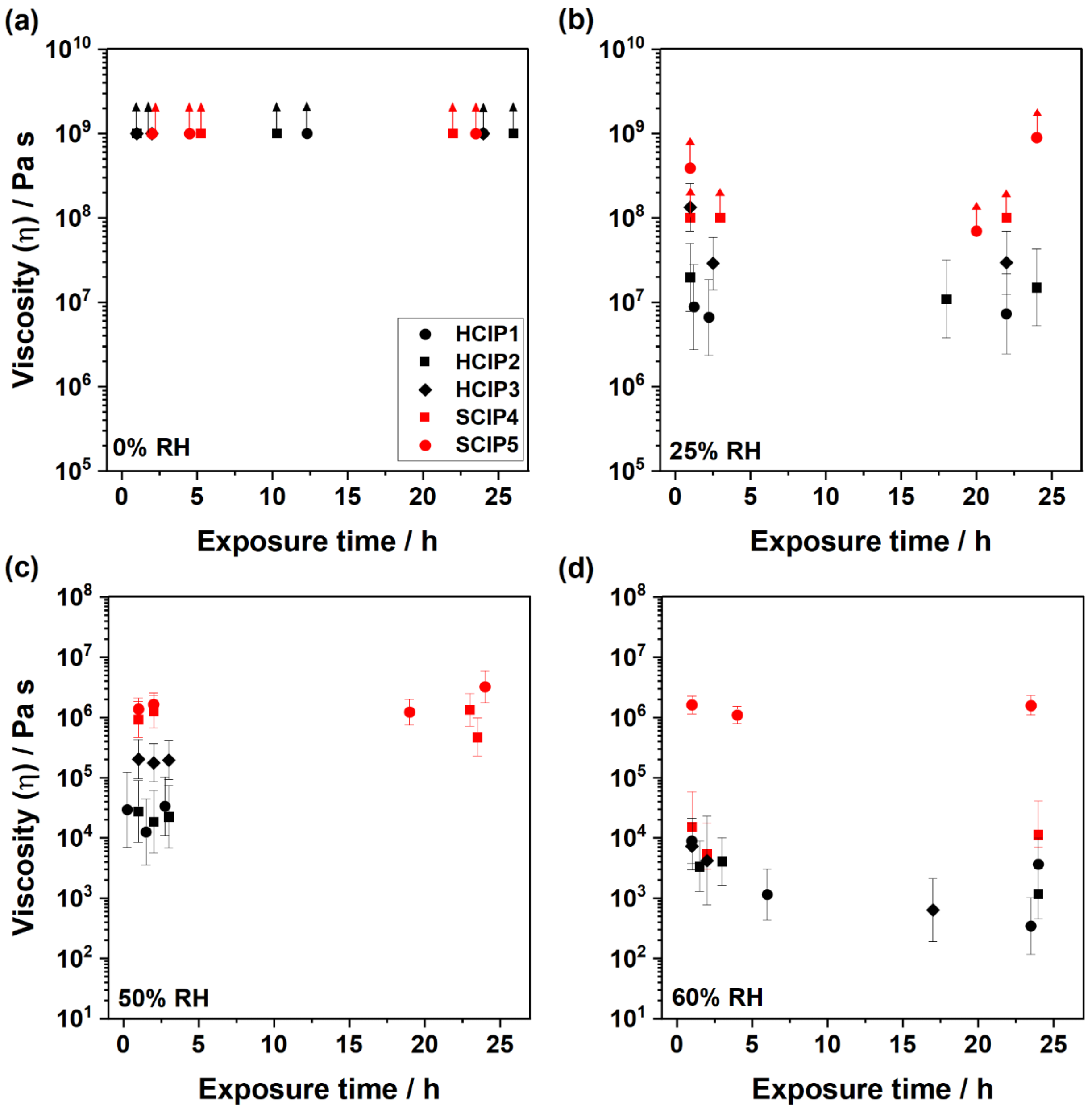

Figure S6: The dependence of viscosity on conditioning time for all SOA types. A poke-flow experiment was performed at various conditioning times during which particles were exposed to an air flow up to 24 h at (a) 0%, (b) 25%, (c) 50%, and (d) 60% RH to ensure that the particles were at equilibrium. Black points correspond to those of healthy plant SOA and red points correspond to stressed plant SOA. Upward arrows indicate lower limits to viscosity.

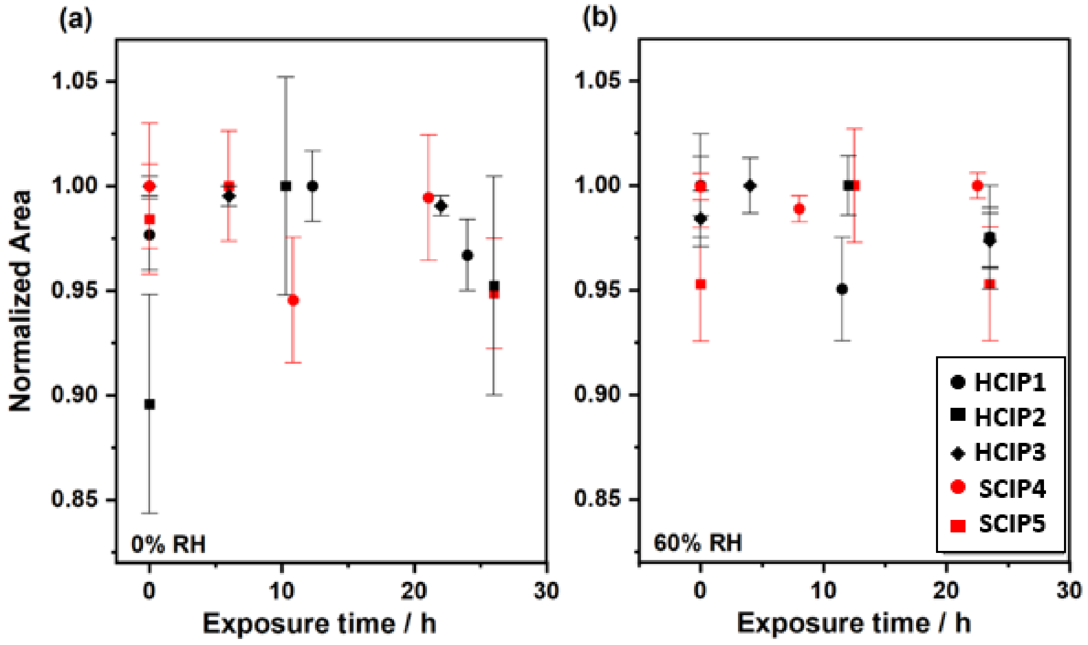

Figure S7: Particle evaporation tests performed within the flow cell of a poke-flow experiment. The area of a SOA particle was monitored for each SOA type over the course of up to 27 h. The particles were exposed to an air flow at (a) ≈ 0% RH and (b) 60% RH within the flow cell, and their area was tracked with the microscope. Error bars correspond to the standard deviation of repeated measurements of the area of the particle at a given time point. Black points correspond to those of healthy plant SOA and red points correspond to stressed plant SOA.

Table S3. Percent of total VOC identified with TD-GC-MS for each experiment. P-values were determined using a two-tailed, Student's t-test for the comparison of the average healthy (HCIP1-3) and stressed (SCIP4-5) trees.

**VOC Name**	**HCIP1 (% total)**	**HCIP2 (% total)**	**HCIP3 (% total)**	**SCIP4 (% total)**	**SCIP5 (% total)**	**p-value**
α-pinene	77.99	82.49	80.00	69.43	78.02	0.17
β-pinene	7.26	8.89	7.56	7.04	14.49	0.39
d-limonene	2.91	1.58	3.16	3.49	2.22	0.73
β-myrcene	1.05	5.16	0.25	2.51	1.30	0.39
camphene	2.15	0.50	1.84	2.21	0.52	0.89
Other MT	7.97	0.74	6.35	10.83	1.08	0.85
Oxy-T	0.59	0.44	0.81	2.44	0.30	0.42
caryophyllene	0.04	0.07	0.01	0.21	0.10	0.10
Stress SQT	0.02	0.01	-	1.17	1.93	0.01*
Other SQT	0.01	0.12	0.01	0.66	0.05	0.27
Total MT	99.33	99.35	99.17	95.52	97.62	
Total OMT	0.59	0.44	0.81	2.44	0.30	
Total SQT	0.08	0.21	0.02	2.05	2.08	

*denotes p-values that are statistically significant assuming a threshold of significance of $p \leq 0.01$.

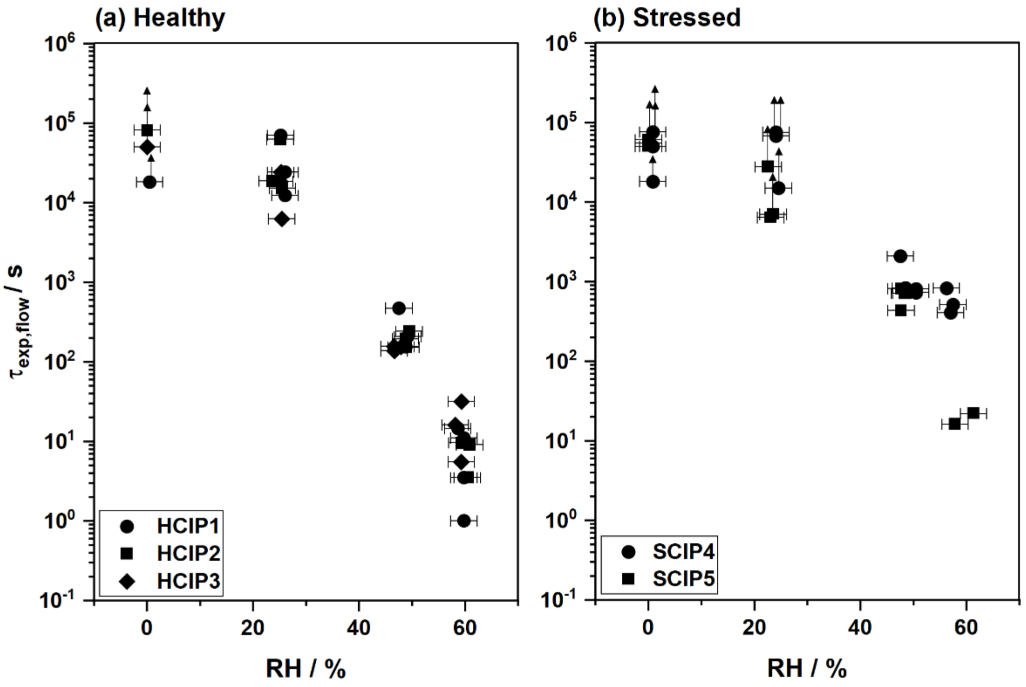

Figure S8: Experimental flow times ($\tau_{exp,flow}$) as a function of relative humidity of poke-flow experiments obtained at room temperature (292 K). Shown are the experimental flow times from each individual poke-flow measurement. These results were used to determine upper and lower limits to the viscosity for each individual measurement. These upper and limits to the viscosity were then combined to give viscosity as a function of RH for each individual tree (Figure S9 and Figure 2). Panel (a) corresponds to those of healthy plant SOA and panel (b) corresponds to those of stressed plant SOA. The error bars in the x-direction correspond to the error in relative humidity from the measurement of dewpoints using a chilled mirror hygrometer. Upward arrows correspond to lower limits, and downwards arrows correspond to upper limits.

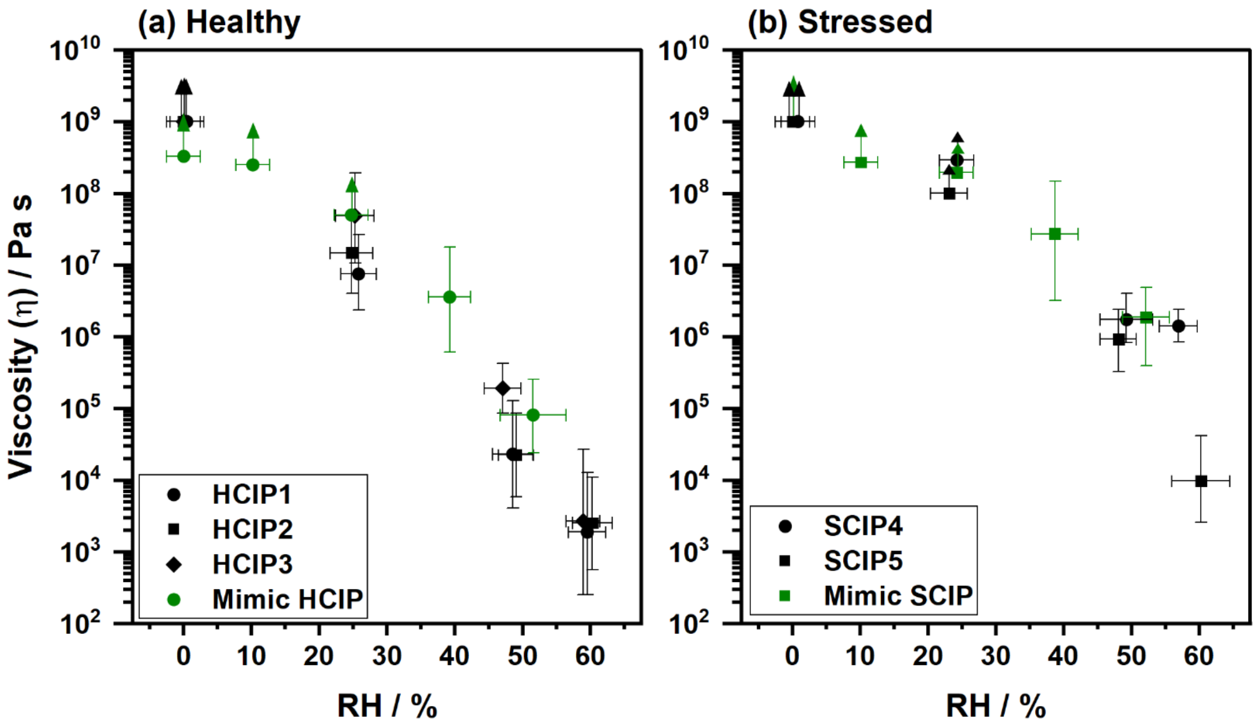

Figure S9: Viscosity as a function of RH of poke-flow experiments obtained at room temperature (292 K). Panel (a) corresponds to those of healthy plant SOA and panel (b) corresponds to those of stressed plant SOA. Green circles (hp-proxy SOA) correspond to data from Smith et al. (2021) and Maclean et al. (2021),[26,27] green squares (sp-proxy SOA) correspond to data from Smith et al. (2021).[26]

References

(1) Faiola, C. L.; Buchholz, A.; Kari, E.; Yli-Pirilä, P.; Holopainen, J. K.; Kivimäenpää, M.; Miettinen, P.; Worsnop, D. R.; Lehtinen, K. E. J.; Guenther, A. B.; Virtanen, A. Terpene Composition Complexity Controls Secondary Organic Aerosol Yields from Scots Pine Volatile Emissions. *Sci Rep* **2018**, *8* (1), 3053. https://doi.org/10.1038/s41598-018-21045-1.

(2) Nakao, S.; Tang, P.; Tang, X.; Clark, C. H.; Qi, L.; Seo, E.; Asa-Awuku, A.; Cocker, D. Density and Elemental Ratios of Secondary Organic Aerosol: Application of a Density Prediction Method. *Atmos Environ* **2013**, *68*, 273–277. https://doi.org/10.1016/J.ATMOSENV.2012.11.006.

(3) Malloy, Q. G. J.; Nakao, S.; Qi, L.; Austin, R.; Stothers, C.; Hagino, H.; Cocker, D. R. Real-Time Aerosol Density Determination Utilizing a Modified Scanning Mobility Particle Sizer—Aerosol Particle Mass Analyzer System. *Aerosol Science and Technology* **2009**, *43* (7), 673–678. https://doi.org/10.1080/02786820902832960.

(4) Eddingsaas, N. C.; Loza, C. L.; Yee, L. D.; Chan, M.; Schilling, K. A.; Chhabra, P. S.; Seinfeld, J. H.; Wennberg, P. O. Atmospheric Chemistry and Physics α-Pinene Photooxidation under Controlled Chemical Conditions-Part 2: SOA Yield and Composition in Low-and High-NO x Environments. *Atmos. Chem. Phys* **2012**, *12*, 7413–7427. https://doi.org/10.5194/acp-12-7413-2012.

(5) Kiland, K. J.; Marroquin, K. L.; Smith, N. R.; Xu, S.; Nizkorodov, S. A.; Bertram, A. K. A New Hot-Stage Microscopy Technique for Measuring Temperature-Dependent Viscosities of Aerosol Particles and Its Application to Farnesene Secondary Organic Aerosol. https://doi.org/10.5194/amt-2022-151.

(6) Kiland, K. J.; Marroquin, K. L.; Smith, N. R.; Xu, S.; Nizkorodov, S. A.; Bertram, A. K. A New Hot-Stage Microscopy Technique for Measuring Temperature-Dependent Viscosities of Aerosol Particles and Its Application to Farnesene Secondary Organic Aerosol. *Atmos Meas Tech* **2022**, *15* (19), 5545–5561. https://doi.org/10.5194/AMT-15-5545-2022.

(7) Faiola, C. L.; Pullinen, I.; Buchholz, A.; Khalaj, F.; Ylisirniö, A.; Kari, E.; Miettinen, P.; Holopainen, J. K.; Kivimäenpää, M.; Schobesberger, S.; Yli-Juuti, T.; Virtanen, A. Secondary Organic Aerosol Formation from Healthy and Aphid-Stressed Scots Pine Emissions. *ACS Earth Space Chem* **2019**, *3* (9), 1756–1772. https://doi.org/10.1021/acsearthspacechem.9b00118.

(8) Demond, A. H.; Lindner, A. S. Estimation of Interfacial Tension between Organic Liquids and Water. *Environ Sci Technol* **1993**, *27* (12), 2318–2331. https://doi.org/10.1021/es00048a004.

(9) Gray Bé, A.; Upshur, M. A.; Liu, P.; Martin, S. T.; Geiger, F. M.; Thomson, R. J. Cloud Activation Potentials for Atmospheric α-Pinene and β-Caryophyllene Ozonolysis Products. *ACS Cent Sci* **2017**, *3* (7), 715–725. https://doi.org/10.1021/acscentsci.7b00112.

(10) Hritz, A. D.; Raymond, T. M.; Dutcher, D. D. A Method for the Direct Measurement of Surface Tension of Collected Atmospherically Relevant Aerosol Particles Using Atomic Force Microscopy. *Atmos Chem Phys* **2016**, *16* (15), 9761–9769. https://doi.org/10.5194/acp-16-9761-2016.

(11) Gorkowski, K.; Donahue, N. M.; Sullivan, R. C. Aerosol Optical Tweezers Constrain the Morphology Evolution of Liquid-Liquid Phase-Separated Atmospheric Particles. *Chem* **2020**, *6* (1), 204–220. https://doi.org/10.1016/j.chempr.2019.10.018.

(12) Churaev, N. V; Sobolev, V. D.; Somov, A. N. Slippage of Liquids over Lyophobic Solid Surfaces. *J Colloid Interface Sci* **1984**, *97* (2), 574–581. https://doi.org/10.1016/0021-9797(84)90330-8.

(13) Zhu, L.; Attard, P.; Neto, C. Reconciling Slip Measurements in Symmetric and Asymmetric Systems. *Langmuir* **2012**, *28* (20), 7768–7774. https://doi.org/10.1021/la301040d.

(14) Baudry, J.; Charlaix, E.; Tonck, A.; Mazuyer, D. Experimental Evidence for a Large Slip Effect at a Nonwetting Fluid-Solid Interface. *Langmuir* **2001**, *17* (17), 5232–5236. https://doi.org/10.1021/la0009994.

(15) Joseph, P.; Tabeling, P. Direct Measurement of the Apparent Slip Length. *Phys Rev E Stat Nonlin Soft Matter Phys* **2005**, *71* (3), 1–4. https://doi.org/10.1103/PhysRevE.71.035303.

(16) Vinogradova, O. I.; Koynov, K.; Best, A.; Feuillebois, F. Direct Measurements of Hydrophobic Slippage Using Double-Focus Fluorescence Cross-Correlation. *Phys Rev Lett* **2009**, *102* (11), 1–4. https://doi.org/10.1103/PhysRevLett.102.118302.

(17) Tretheway, D. C.; Meinhart, C. D. Apparent Fluid Slip at Hydrophobic Microchannel Walls. *Physics of Fluids* **2002**, *14* (3). https://doi.org/10.1063/1.1432696.

(18) McBride, S. P.; Law, B. M. Viscosity-Dependent Liquid Slip at Molecularly Smooth Hydrophobic Surfaces. *Phys Rev E Stat Nonlin Soft Matter Phys* **2009**, *80* (6), 2–5. https://doi.org/10.1103/PhysRevE.80.060601.

(19) Jing, D.; Bhushan, B. Boundary Slip of Superoleophilic, Oleophobic, and Superoleophobic Surfaces Immersed in Deionized Water, Hexadecane, and Ethylene Glycol. *Langmuir* **2013**, *29* (47), 14691–14700. https://doi.org/10.1021/la4030876.

(20) Craig, V. S. J.; Neto, C.; Williams, D. R. M. Shear-Dependent Boundary Slip in an Aqueous Newtonian Liquid. *Phys Rev Lett* **2001**, *87* (5), 54504-1-54504–4. https://doi.org/10.1103/PhysRevLett.87.054504.

(21) Cottin-Bizonne, C.; Jurine, S.; Baudry, J.; Crassous, J.; Restagno, F.; Charlaix, É. Nanorheology: An Investigation of the Boundary Condition at Hydrophobic and Hydrophilic Interfaces. *European Physical Journal E* **2002**, *9* (1), 47–53. https://doi.org/10.1140/epje/i2001-10112-9.

(22) Cottin-Bizonne, C.; Cross, B.; Steinberger, A.; Charlaix, E. Boundary Slip on Smooth Hydrophobic Surfaces: Intrinsic Effects and Possible Artifacts. *Phys Rev Lett* **2005**, *94* (5), 1–4. https://doi.org/10.1103/PhysRevLett.94.056102.

(23) Cho, J. H. J.; Law, B. M.; Rieutord, F. Dipole-Dependent Slip of Newtonian Liquids at Smooth Solid Hydrophobic Surfaces. *Phys Rev Lett* **2004**, *92* (16), 1–4. https://doi.org/10.1103/PhysRevLett.92.166102.

(24) Bhushan, B.; Wang, Y.; Maali, A. Boundary Slip Study on Hydrophilic, Hydrophobic, and Superhydrophobic Surfaces with Dynamic Atomic Force Microscopy. *Langmuir* **2009**, *25* (14), 8117–8121. https://doi.org/10.1021/la900612s.

(25) Chesna, J. W.; Wiedmaier, B. F.; Wang, J.; Samara, A.; Leach, R. K.; Her, T. H.; Smith, S. T. Aerial Wetting Contact Angle Measurement Using Confocal Microscopy. *Meas Sci Technol* **2016**, *27* (12). https://doi.org/10.1088/0957-0233/27/12/125202.

(26) Smith, N. R.; Crescenzo, G. v.; Huang, Y.; Hettiyadura, A. P. S.; Siemens, K.; Li, Y.; Faiola, C. L.; Laskin, A.; Shiraiwa, M.; Bertram, A. K.; Nizkorodov, S. A. Viscosity and Liquid–Liquid Phase Separation in Healthy and Stressed Plant SOA. *Environmental Science: Atmospheres* **2021**, 140–153. https://doi.org/10.1039/d0ea00020e.

(27) Maclean, A. M.; Li, Y.; Crescenzo, G. v.; Smith, N. R.; Karydis, V. A.; Tsimpidi, A. P.; Butenhoff, C. L.; Faiola, C. L.; Lelieveld, J.; Nizkorodov, S. A.; Shiraiwa, M.; Bertram, A. K. Global Distribution of the Phase State and Mixing Times within Secondary Organic Aerosol Particles in the Troposphere Based on Room-Temperature Viscosity Measurements. *ACS Earth Space Chem* **2021**, *5* (12), 3458–3473. https://doi.org/10.1021/acsearthspacechem.1c00296.
